# Protective effect of *Nigella sativa *and thymoquinone on serum/glucose deprivation-induced DNA damage in PC12 cells

**Published:** 2012

**Authors:** Beheshteh Babazadeh, Hamid Reza Sadeghnia, Elham Safarpour Kapurchal, Heydar Parsaee, Sima Nasri, Zahra Tayarani-Najaran

**Affiliations:** 1 *Department of Biology, Payame Noor University (PNU), Tehran, I. R. Iran*; 2 *Pharmacological Research Center of Medicinal plants, Department of Pharmacology, School of Medicine, Mashhad University of Medical Sciences (MUMS), Mashhad, I. R. Iran*; 3 *Department of Biology, Qom Branch, Islamic Azad University, Qom, I. R. Iran*

**Keywords:** Serum/glucose deprivation, DNA damage, PC12 cells, Thymoquinone, * Nigella sativa*

## Abstract

**Objective:** The discovery and development of natural products with potent antioxidant properties has been one of the most interesting and promising approaches in the search for treatment of CNS injuries. The most significant consequence of the oxidative stress is thought to be the DNA modifications, which can become permanent via the formation of mutations and other types of genomic instability resulting cellular dysfunction. Serum/glucose deprivation (SGD) has served as an excellent in vitro model for the understanding of the molecular mechanisms of neuronal damage during ischemia and for the development of neuroprotective drugs against ischemia-induced brain injury*. Nigella sativa (N. sativa) *seeds and thymoquinone (TQ), its most abundant constituent, have been shown to possess anti-inflammatory, antioxidant, chemopreventive and anti-neoplastic effects both in vitro and in vivo. Therefore, in this study we investigated genoprotective effects of *N. sativa* and TQ on DNA damage of PC12 cells under SGD condition.

**Materials and Methods:** PC12 cells were cultured in DMEM medium containing 10% (v/v) fetal bovine serum, 100 units/ml penicillin, and 100 µg/ml streptomycin. Initially cells were pretreated with different concentrations of *N. sativa *extract (NSE), (10, 50, 250 µg/ml) and TQ (1, 5, 10 µg/ml) for 6 h and then deprived of serum/glucose (SGD) for 18 h. The alkaline comet assay was used to evaluate the effect of these compounds on DNA damage following ischemic insult. The amount of DNA in the comet tail (% tail DNA) was measured as an indicator of DNA damage.

**Results: **A significant increase in the % tail DNA was seen in nuclei of cells following SGD induced DNA damage (p<0.001). In the control groups, no significant difference was found in the % tail DNA between NSE- or TQ-pretreated and vehicle-pretreated PC12 cells (p>0.05). NSE and TQ pretreatment resulted in a significant decrease in DNA damage following ischemic insult (p<0.001). This suppression of DNA damage by NSE and TQ was found to be dose-dependent.

**Conclusion**: These data indicate that NSE and TQ have a genoprotective property, as revealed by the comet assay, under SGD condition in PC12 cells.

## Introduction

Reactive oxygen species (ROS) is presumably involved in pathogenesis of ischemia-induced neuronal cell damage as well as neurodegenerative disorders. Oxidative stress results in neuronal cell death and activation of apoptotic process (Collins et al., 1998 ▶; Morocz et al., 2002 ▶). Also oxidative damage to DNA has been identified as a useful index of oxidative stress, and shown to be elevated in patients with neurodegenerative disorders such as Alzheimer’s disease (Kadioglu et al., 2004 ▶; Reardon et al., 1997 ▶; Lindahl, 1993 ▶) A well-defined cell system for in vitro studies of SGD-evoked neuronal injury can be provided by the rat pheochromocytoma (PC12) cell line (Hillion et al., 2005 ▶). Serum/glucose deprivation (SGD) has served as an excellent in vitro model for the understanding of the molecular mechanisms of neuronal damage during brain ischemia and for the development of neuroprotective drugs against ischemia-induced brain injury (Amantea et al., 2009 ▶; Behl and Moosmann, 2002 ▶; Chu et al, 2008 ▶). Cultured neural cells can undergo apoptosis in response to component stimuli of ischemia, such as hypoxia, serum and nutrient deprivation, and metabolic stress (Hillion et al., 2005 ▶). Many antioxidants can reduce neuronal cell damage induced by oxidative stress through augmenting endogenous defense capacities (Mousavi et al., 2010 ▶).

*Nigella sativa *L. (family Ranunculaceae), commonly known as black seed or black cumin, is an annual plant that has been traditionally used as a natural remedy for a number of illnesses (Ali and Blunden, 2003 ▶). Notable pharmacological properties such as antioxidative (Ochiaia et al., 2004 ▶), immunomodulation (Ali and Blunden, 2003 ▶), anti-inﬂammatory (Houghton et al., 1995 ▶), neuroprotective, anti-ischemic, antiepileptic and anxiolytic effects have been reported for NSE or its constituents (Kanter et al., 2006 ▶; Hosseinzadeh et al., 2007; Ilhan et al., 2005; Gilhotra and Dhingra, 2011).  ▶Many of the pharmacological activities mentioned above have been attributed to quinone constituents in the seed, especially thymoquinone (TQ) (Salem, 2005 ▶). TQ has been reported to exhibit antioxidant (Badary et al., 1999 ▶; Badary et al., 2003), anti-inflammatory, neuroprotective, anti-ischemic and chemopreventive effects (Burits and Bucar, 2000 ▶; Ostling and Johanson, 1984 ▶). In this study, we sought to determine the possible protective effects of hydroalcoholic extract of *N. sativa *seed and TQ against cell death and DNA damage in PC-12 cells under SGD condition. 

Single cell gel electrophoresis, also known as the ‘‘Comet assay” has been widely used to detect DNA lesions such as strand breaks, alkali-labile sites, DNA cross-linking, and incomplete excision repair sites. This technique has been shown to be very sensitive and is therefore useful for the detection of genetic damage at the individual cell level (Singh et al., 1988; Kassie et al., 2000 ▶). 

## Materials and Methods

**Reagents**

Chemicals were obtained from the following sources:* N. sativa *seeds were authenticated by Pharmacognosy Department, School of Pharmacy, MUMS (Mashhad, I.R. Iran). γ-tocopherol (sigma), Dulbecco’s modified eagle’s medium (DMEM) and fetal bovine serum (FBS) were obtained from Gibco. Low melting point (LMP) agarose from Biogen (Mashhad, I.R. Iran); normal melting point (NMP) agarose from Fermentas (Glen Burnie, MD), and sodium hydroxide (NaOH ), sodium chloride (NaCl), ethylene diamine tetraacetic acid disodium salt (Na_2_EDTA), Tris (hydroxymethyl) aminomethane (Trizmabase), toctylphenoxypoly- ethoxyethanol (Triton X-100), dimethylsulfoxide (DMSO), sodiumlauroylsarcosinate (sarkosyl, SLS), ethidium bromide, and methanol from Merck (Darmstadt, Germany). LMP and NMP agarose were diluted in physiological saline to 0.5% and 1%, respectively, and γ-tococherol was dissolved in DMSO at 1 mg/ml concentration, and stored at -70 °C until use.

**Preparation of the** **NSE**

*N. sativa* seeds were collected from Gonabad region (northeast of Iran) and authenticated by herbarium of Ferdowsi University (Mashhad, Iran; voucher specimen No. 293-0303-1). 

The seeds of* N. sativa* were washed, dried, and crushed to a powder with an electric microniser. The powdered seeds (100 g) were extracted in a Soxhlet extractor with ethanol (70%) and the resulting extract was dried and kept at -20 ºC until use (32% yield). 

Stock solution of NSE and TQ were prepared in DMSO and suitable working concentrations were made from the stock using complete medium

**Cell culture**

PC12 cells were purchased from Pasteur Institute (Tehran, Iran). Cells were maintained at 37°C in a humidified atmosphere (90%) containing 5% CO2. Cells were cultured in high-glucose Dulbecco’s modified Eagle’s medium (DMEM) (4.5 g/l) with 10% (v/v) fetal bovine serum, 100 U/ml penicillin, and 100 U/ml streptomycin. For the experiments, PC12 cells were seeded overnight and then cells were pretreated (6 h) with NSE (10, 50, 250 µg/ml) and TQ (1, 5, 10 µg/ml) and subjected to SGD for 18 h. For comet assay, cells were seeded at 1000,000 on to 6-well culture plates. γ-Tocopherol was tested as positive control. 

**Comet assay**

The in-vitro alkaline SCGE assay was conducted based on the method described previously (Hosseinzadeh et al., 2008 ▶). One hundred microliters of NMP agarose was quickly layered on conventional slides, the slides were covered with a cover slip, and then the slides were placed on ice to allow agarose to gel. Five microliters of the nucleus suspension, prepared as above, was mixed with 75 ml LMP agarose, and the mixture was quickly layered over the NMP agarose layer after removal of the cover slip. Finally, another layer of LMP agarose was added on top. The slides were immersed immediately in a chilled lysing solution (pH=10) made up of 2.5 M NaCl, 100 mM Na2EDTA, 10mM Trizma, 1% sarkosyl, 10% DMSO, and 1% Triton X-100, and kept at 0°C in the dark overnight. Then, the slides were placed on a horizontal gel electrophoresis platform and covered with a prechilled alkaline solution made up of 300 mM NaOH and 1 mM Na_2_EDTA (pH=13). 

They were left in the solution in the dark at 0°C for 40 min, and then electrophoresed at 0°C in the dark for 30 min at 25 V and approximately 300 mA. The slides were rinsed gently three times with 400 mM Trizma solution (adjusted to pH 7.5 by HCl) to neutralize the excess alkali, stained with 50 ml of 20 µg/ml ethidium bromide, and covered with a cover slip.

**Comet analysis**

One hundred nuclei per slide from each group (50 nuclei on one slide) were examined and photographed through a fluorescence microscope (Nikon, Kyoto, Japan) at 400x magnification equipped with an excitation filter of 520-550 nm and a barrier filter of 580 nm. Undamaged cells resemble an intact nucleus without a tail, and damaged cells have the appearance of a comet. The amount of DNA in the comet tail (% tail DNA), which is an estimate of DNA damage, was measured using a computerized image analysis software (CASP software). 

**Statistical analysis**

All results were expressed as mean±SEM. Statistical differences between groups were analyzed by one way analysis of variance (ANOVA) with subsequent Tukey’s tests. A probability level of p<0.05 was considered significant statistically. 

## Results

In this study, % tail DNA was measured as an indicator of DNA damage. In control groups, no significant difference was found in the % tail DNA between NES- or TQ-pretreated and vehicle-pretreated PC12 cells (p>0.05) (Figures 2, 3). The results showed that *N. sativa *and TQ could decrease DNA fragmentation (DF) of cultured PC12 cells in a dose-dependent manner compared to negative control under SGD condition (p<0.001).

**Figure 1:**
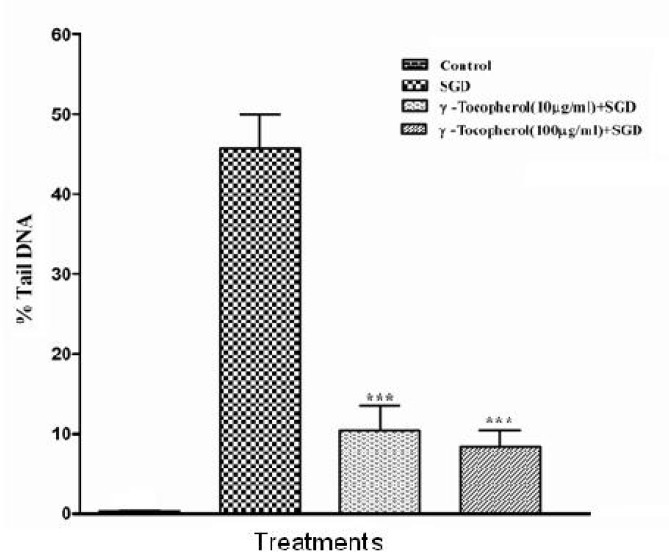
%Tail DNA induced by serum/glucose deprivation (SGD) in PC12 cells after 18 h. Cells were pretreated with different concentrations of γ-tocopherol (10, 100 μg/ml) for 6 hours. Control cells were exposed to normal media (DMEM) containing 0.1% dimethyl sulfoxide (DMSO). Pretreatment with γ-tocopherol (10, 100 µg/mL) significantly decreased the % tail DNA induced by SGD (about 76% and 84%, respectively). All data were represented as the means±SEM of three independent experiments. ***p<0.001, as compared with SGD

**Figure 2:**
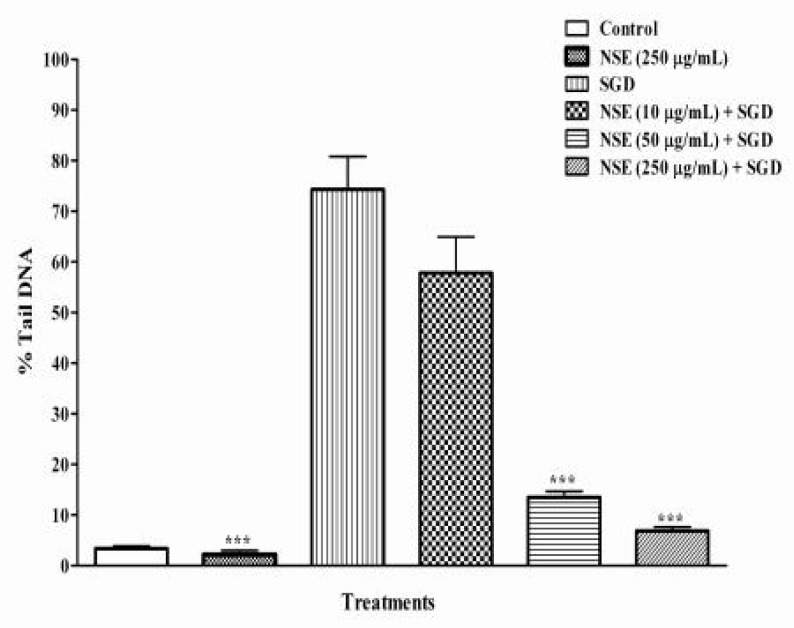
%Tail DNA induced by serum/glucose deprivation (SGD) in PC12 cells after 18 h. Cells were pretreated with different concentrations of NSE for 6 hours. Control cells were exposed to normal media (DMEM) containing 0.1% dimethyl sulfoxide (DMSO) with 250 μg/ml of NSE. Pretreatment with 250 μg/ml of NS significantly decreased the %Tail DNA induced by SGD. All data were represented as the means±SEM of three independent experiments. ***p<0.001, as compared with SGD.

**Figure 3:**
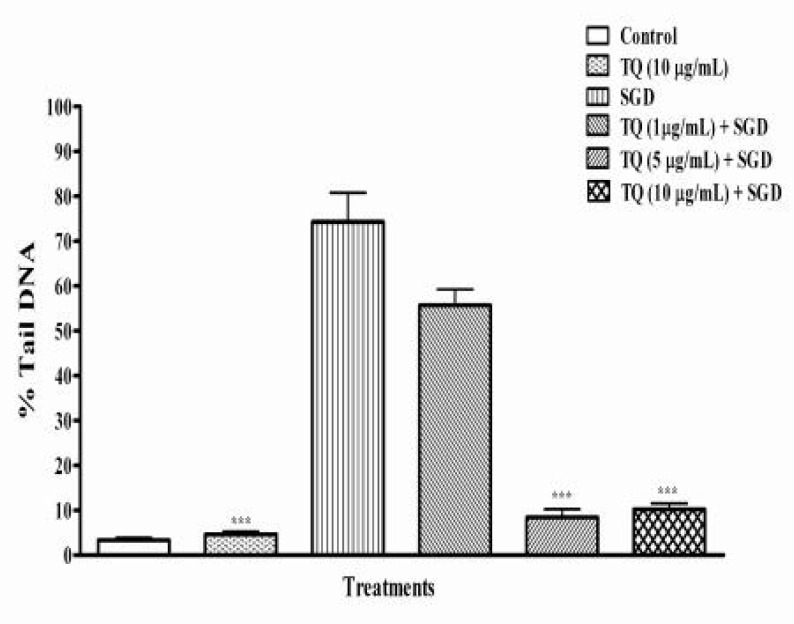
%Tail DNA induced by serum/glucose deprivation (SGD) in PC12 cells after 18 h. Cells were pretreated with different concentrations of thymoquinone (TQ) for 6 hours. Control cells were exposed to normal media (DMEM) containing 0.1% dimethyl sulfoxide (DMSO) with 10 μg/ml of TQ. TQ (between 5-10 µg/ml) significantly reduced DNA damage induced by SGD. All data were represented as the means±SEM of three independent experiments. ***p< 0.001, as compared with SGD

A significant decrease in SGD-induced DF was seen following pretreatment with high dose of NES (89%, 250 µg/mL) and TQ (87%, 10 µg/mL), respectively (Figures 2, 3). As a positive control, γ-tocopherol protected PC12 cells against SGD-induced DF, as pretreatment with 100 and 10 µg/ml of γ-tocopherol significantly decreased the DF when compared to with SGD group. Pretreatment with vitamine E 10 and 100 µg/mL resulted in 76% and about 84% reduction of DF, respectively (Figure 1). We found that *N. sativa* and TQ exhibited reduction of DF against SGD-induced DNA damage in PC-12 cells (only TQ 1 µg/ml). 

## Discussion

The basis of many neurological and neurodegenerative disorders such as ischemia-reperfusion injury, seizure, Parkinson’s and Alzheimer’s disease, at least partially, is the generation of free radicals (Gilgun et al., 2002 ▶). A marked increase in lipid peroxidation and generation of oxidative DNA damage following transient middle cerebral artery occlusion has been reported in rats (Choi et al., 2004 ▶). In this study we used serum/glucose deprivation (SGD) model for induction of DNA damage in PC12 cells. SGD is defined as an in vitro model of the pathological process of cerebral ischemia (Hillion et al., 2005 ▶, Mousavi et al., 2010 ▶). The results of the preceding investigation suggest that intracellular ROS production is significantly increased following SGD (Mousavi et al., 2010 ▶). The result of ROS formation is damage to an array of biomolecules found in cells, including membrane lipids, proteins and nucleic acids (Datta and Namasivayam, 2003 ▶). It has been shown that oxidative stress following production of reactive oxygen species may play an important role in DF and in apoptotic cell death caused by Beta-amyloid (Cecchi et al., 2007 ▶). 

The antioxidants are certainly among the most promising therapeutic class for the treatment of ischemic strokes, and this may probably be, in part, due to the fact that their therapeutic window may be longer than that of other strategies (Margaill et al., 2005 ▶). Therefore, the discovery and development of potent antioxidant agents has been one of the most interesting and promising approaches in the search for treatment of CNS injury (Gilgun et al., 2002 ▶). 

In this study, we showed that serum/glucose deprivation in PC12 cells induced DF after 18 h compared to the controls via comet assay. This observation is in agreement with previous studies, which suggest that serum and glucose deprivation, in combination with the addition of 2-deoxyglucose, induces time-dependent apoptosis in cultured neonatal rat cardiac myocytes, as evidenced by decrease in nuclear morphology and internucleosomal DF (Bialik et al, 1999) ▶. Using the comet assay, we showed that NSE and thymoquinone (between 5-10 µg/ml) suppressed SGD-induced DF in PC12 cells. 

It has been shown that α-tocopherol plays an important role in reducing membrane damage caused by excessive ROS production, and then reducing lipid peroxidation and lowering the expression of apoptosis genes by reduced DF. Also neuroprotective effects of α-tocopherol were mediated by its antioxidant activity (Jeong et al., 2009; Paul, 2007 ▶). Therefore, in this study we compared the attenuation of DF by *N. sativa* and TQ with γ-tocopherol, under SGD condition. 

Our results demonstrated that* N. sativa* and thymoquinon exhibited reduction of DF even more than γ-tocopherol, which may be due to antioxidant and anti-apoptotic effects of *N. sativa* and TQ. 

It is assumed that these probable anti-apoptogenic effects of *N. sativa* and TQ may be mediated by one or more of the following mechanisms: Antioxidant activity, immunomedulatory action and genoprotective effects (Mousavi et al., 2010 ▶; Burits and Bucar; 2000 ▶; Sethi et al., 2008 ▶; Rastogi et al., 2010). 

According to the previous studies, *N. sativa* oil protects lipids against free-radical damage (Burits and Bucar, 2000 ▶). Decreased tissue malondialdehyde (MDA), protein carbonyl levels and prevented inhibition of superoxide dismutase (SOD), glutathione peroxidase (GSH-Px), and catalase (CAT) enzyme activities following experimental spinal cord injury in rats were seen following treatment with *N. sativa* (Kanter et al., 2006 ▶). 

It has been shown that TQ inhibits non-enzymatic lipid peroxidation in liposomes (Burits and Bucar 2000 ▶). Another study showed that neuroprotective action of *N. sativa* oil following pentylenetetrazol (PTZ)-induced seizures in mice may correlate with its ability to inhibit excessive reactive oxygen species (ROS) formation (Ilhan et al., 2005). Pretreatment with TQ and *N. sativa* oil significantly decreased MDA level as compared with ischemic group during global cerebral ischemia-reperfusion injury in rat hippocampus (Hosseinzadeh et al., 2007). Another study has also indicated that neuroprotective effects of TQ and NSE in STZ induced diabetic rats are attributed to its antioxidant activity (Kanter, 2008 ▶). *N. sativa* oil and its fractions, hexane fraction (HF), ethyl acetate fraction (EAF) and water fraction (WF), prevented beta amyloid (Aβ)-induced cell death in primary rat cerebellar granule neurons via antioxidant properties (**Rastogi et al., 2010**). Recently, we showed that *N. sativa *and TQ protect PC12 cells against SGD-induced cell death through antioxidant mechanisms (Mousavi et al., 2010 ▶). 

Recently, the results obtained from the different experimental systems suggested the radioprotective ability of ethanolic extract of *N. sativa* involving prevention of radiation-induced oxidative damage of DNA (Rastogi et al., 2010). 

According to the previous experiments, TQ clearly inhibits NF-kB activation, which makes it a potentially effective suppressor of apoptosis, inflammation, tumor cell proliferation, and angiogenesis (Sethi et al., 2008 ▶). A recent study indicated that *N. sativa* prevents formaldehyde induced neuronal injury, through reduction of the caspase 3 immunoreactivity of degenerating neurons, and eventually apoptosis in frontal cortex (Kanter, 2010 ▶).

In conclusion, our study demonstrated that SGD significantly increased DNA fragmentation which is prevented by pretreatment with *N. sativa* and TQ. 
